# Evolutionary game analysis of polluting NIMBY facilities reconstruction based on public participation behavior

**DOI:** 10.1371/journal.pone.0276272

**Published:** 2022-10-17

**Authors:** Hui Zhao, Mengran Zhang, Weihan Wang

**Affiliations:** School of Management Engineering, Qingdao University of Technology, Qingdao, China; Shandong University of Science and Technology, CHINA

## Abstract

With the advancement of urbanization and the expansion of urban areas, NIMBY (not in my back yard) environmental public facilities are increasing day by day. It is meaningful to incorporate public participation into the regulatory process for the existing pollution NIMBY facility enterprises. Through the establishment of the tripartite game model of local government, polluting NIMBY facility enterprises and the public, the evolution stability analysis and simulation analysis of their strategies are carried out, and the Pareto optimal solution is obtained. The results show that: The strategy choices of the players of the three-party game are different under different stability conditions. The system can be broken out of the bad state by increasing government punishment, local governments strictly controlling the potential profits, the potential losses of polluting enterprises not rebuilding, the long-term public benefits and reducing the cost of public participation, etc., and the three-party common governance mode can be formed. The strategy evolution speed of a player in a three-party game is affected by his own strategy choice proportion and the strategy choice proportion of the other two players, but no matter how the strategy choice proportion of the player in a three-party game changes, it will not change the final game result. On the basis of comprehensive analysis, a series of relevant suggestions are put forward from the three aspects of government, enterprises and the public, so as to provide certain reference for the design of the public participation system of polluting NIMBY facilities.

## 1. Introduction

With the advance of global urbanization and the expansion of urban population, cities have increased the demand for various environmental services. Meanwhile, NIMBY-type environmental public facilities are also increasing. With the expansion of urbanization areas, NIMBY-type public facilities built earlier are gradually surrounded by residential areas or commercial service areas.NIMBY facilities [1] refer to public facilities that serve the majority of people in the region but have negative externality effects [2]. Because of the environmental pollution and threats to residents’ lives and property caused by NIMBY facilities, such as psychological, safety and health hazards are borne by residents near the facilities. NIMBY conflicts are often caused by the dissatisfaction of nearby residents. Meanwhile, the public’s protest methods have changed from relatively gentle means such as banner pulling and sit-in to violent conflicts and other fierce protest methods, which have further developed into mass incidents affecting social stability. If not handled properly, it will not only affect the normal economic development and the stability of social order, but also reduce the credibility of the government. Therefore, the governance of NIMBY facilities has become an important task for the government to build a harmonious society. However, the current government has relatively low capacity to deal with the construction of NIMBY facilities, especially the governance ability of NIMBY public facilities that has been built and then surrounded by residential areas. In order to curb the negative externality effect caused by NIMBY facilities on the surrounding residents, effective measures must be taken to avoid NIMBY protests.

At present, there are mainly two types of research on NIMBY facilities. The first type analyzes the related theories, concepts and concepts of NIMBY from the perspective of different disciplines. For example, causes of NIMBY conflicts are explained from the perspectives of public management, economics, psychology and communication. The second is to study how to avoid the occurrence of NIMBY conflicts. For instance, governance schemes are proposed from the perspectives of law, government governance mechanism and public participation. Most of the former are based on theory and fail to match the new policies related to social governance, which has been out of step with the development of The Times. The latter mostly focus on the early stage of project establishment, ignoring the reconstruction of already built NIMBY facilities. In addition, in terms of research methods, current research methods in NIMBY facilities are relatively simple. Comparatively, there are more theoretical thinking, comparative research and case analysis methods, while there are fewer studies using game theory, interest analysis and quantitative research.

Although game theory has been applied by scholars in the study of NIMBY conflicts, which provides a good theoretical support for solving NIMBY conflicts, it is unrealistic to rely solely on NIMBY enterprises to take the initiative in environmental reconstruction because the development of NIMBY enterprises is profit-oriented. Therefore, it a presses need to deeply study the relationship between relevant stakeholders in the NIMBY conflict in order to alleviate the NIMBY conflict. With the enhancement of residents’ awareness of environmental protection and the pursuit of high quality of living environment, the environmental pollution caused by NIMBY enterprises has attracted more and more attention from the public. Therefore, not only the local government but also the public should participate in the management and supervision of pollution-type NIMBY enterprises. As the ultimate bearer of environmental pollution, the public actively participates in the supervision and governance, on the one hand, it can make up for the defects of the government’s unilateral supervision, on the other hand, it can reduce the occurrence of NIMBY conflicts. Therefore, adding public participation into the supervision and governance of NIMBY enterprises is a necessary measure to relieve NIMBY conflicts. In order to explain the process of environmental protection reconstruction supervision of pollution-type NIMBY enterprises with public participation in a dynamic environment, this paper attempts to analyze the above process with the theory and method of the evolutionary game. Because evolutionary game theory is a theory that combines the analysis of game theory with the analysis of dynamic evolutionary process, it emphasizes a kind of dynamic equilibrium, and takes the group of players as the research object to explain why and how the group reaches this state. The three parties in the game repeatedly make strategic decisions in the dynamic competitive environment, and finally form the evolution and equilibrium of strategic choices. Therefore, the evolutionary game method can clearly describe the decision-making process of the three parties in the regulatory and governance process of NIMBY enterprises.

In order to fill the research gap, this paper selects the research on the reconstruction of existing NIMBY facilities surrounded by residential areas or commercial service areas in the early years. Secondly, the stakeholder evolution model of the reconstruction project of existing polluting NIMBY facility enterprises is constructed by using the evolutionary game method, and the numerical simulation is performed by using Matlab simulation software to verify the model, which has strong explanatory power. Finally, according to the simulation results, some practical suggestions are put forward. This paper makes a contribution to the existing literature by linking the three-party evolutionary game model with the regulation and governance of established pollution-type NIMBY facility enterprises. At the same time, it discusses the possibility of environmental reconstruction of existing NIMBY enterprises from the perspective of stakeholders’ behavior, which provides a new perspective for solving the NIMBY dilemma and makes up for the shortage of relevant theoretical research. In addition, this study illustrates how to choose the strategies of all parties to maximize their own interests under the premise of public participation in supervision and governance, and finally achieve the goal of promoting environmental reconstruction of NIMBY enterprises. Therefore, the research results of this paper can provide reference for policy makers to formulate policies to alleviate NIMBY conflicts.

The remainder of this study is organized as following. The second part reviews the research status of NIMBY facility project. The third part constructs the basic model of this study, establishes an evolutionary game model, and determines the payoff matrix of the game among the three participants: pollution-type NIMBY facility enterprises, local governments, and the public. In the fourth part, game equilibrium analysis, evolutionary stability analysis and simulation analysis are carried out. In the fifth part, the simulation results are discussed and some feasible suggestions are put forward. Finally, the sixth part gives the conclusion and the next step.

## 2. Literature review

### 2.1 NIMBY effect and its influencing path

O ’Hare first coined the term NIMBY(Not In My Backyard,) in 1977 to describe facilities that have a positive effect on society as a whole but may have a negative effect on nearby residents. With the deepening of the study, the researchers found that in addition to the park, a library, most of the public facilities are preventing effect, adjacent scope expands gradually, from the facilities of the surrounding people potentially threatening or merely its psychological discomfort facilities is also believed to belong to adjacent facilities, such as a funeral home and cemetery, crematorium, Even the most popular convenience facilities, such as shopping malls, are also listed as NIMBY facilities due to noise, traffic and other reasons, that is, "all the facilities that will produce negative externalities and make people hate to be adjacent to them", belong to NIMBY facilities.

Based on the analysis and summary of the literature related to NIMBY conflict, researchers believe that the main reasons affecting the occurrence of NIMBY conflict are as follows: First of all, due to the negative externality of NIMBY facilities, people feel that NIMBY facilities may affect their physical health or even pose a serious threat to life and property, which leads to their fear and psychological "sense of deprivation" and "sense of injustice" [3]. At the same time, this negative externality makes it provide necessary output for the society, while its additional potential risk accumulates in a few communities. Such spatial accumulation of risks leads to psychological imbalance and relative deprivation among residents who are forced to bear NIMBY risks [4]. Due to the value deviation in the spatial production of NIMBY facilities, the failure to effectively balance and take into account the interests of all subjects leads to the imbalance of spatial distribution justice, and the failure to ensure the participation of stakeholders leads to the imbalance of spatial process justice, so the positioning and location of NIMBY facilities are prone to opposition from local residents [5]. Secondly, local government officials tend to support the implementation of NIMBY facilities because of their dual economic and political incentives for NIMBY projects in order to achieve the goals of financial growth and tenure performance [6]. However, as the main participant in the construction of NIMBY facilities, the government’s unilateral control mode cannot balance the diversified interest demands. Stakeholder groups have no say in the decision-making process of NIMBY, and it is difficult to participate in decision-making and express opinions through proper channels, so they can only resist the construction of NIMBY facilities through irrational protests [7]. Administrative decisions such as site selection, construction, compensation and governance of NIMBY facilities are dominated by the government. Because planners to confidential project plan, the lack of prior consultation with local residents, and think they have no right to comment in advance [8], led to the adjacent to avoid risk information not publicly or public in a timely manner, not comprehensive, public interests can not get attention and response, information inequality, to both sides information asymmetry. Due to the asymmetric information, the public has no right to speak in the decision-making process of NIMBY projects and cannot interact directly with the government, which leads to public protests and even turns into mass incidents. Finally, as an important subject in NIMBY projects, enterprises take economic interests as the goal in the construction of projects, pursue the maximization of their own interests, do not take the initiative to pay attention to the interests of the surrounding public, and do not take the initiative to improve their living environment and quality of life. In the process of project operation, some pollution-type NIMBY facilities will violate the rules to pursue more interests, which further damages the interests of neighborhood residents. However, when NIMBY conflict occurs, enterprises will not take the initiative to make profits or increase investment to solve the conflict, but rely on the government to take administrative measures to stop it. Such profit-driven nature of enterprises is difficult to build a bridge of trust with the public. Such behavior of enterprises has intensified the conflicts between residents, the government and enterprises.

### 2.2 Research methods of NIMBY problem

NIMBY problem is a multi-scientific interdisciplinary subject. In recent years, scholars from all over the world have conducted rich studies from the perspectives of political science, economics, sociology, psychology and other disciplines, and have shifted from theoretical basic research to process analysis and governance countermeasure research.

Morel found in his research that the main reason for opposing nimby public facilities is that the surrounding people believe that they have been deprived of the right to participate in the decision-making process of project location, and they have a certain sense of inferiority and unfairness due to the construction of facilities around them, which leads to a series of demonstrations and protests against the construction of facilities [9]. Sandman believes that in the decision-making stage, no matter what the reason, as long as the public’s perception of environmental risks of engineering projects is different from the risk assessment of relevant experts, there will be the possibility of public protests and resistance. Therefore, risk communication can be carried out to obtain the consistency of risk understanding between the public and experts and other stakeholders, so as to avoid public protests and resistance [10]. Tao et al. took NIMBY complex as the starting point and constructed the research framework of "expected loss—uncertainty", highlighting that public participation is an important means to resolve conflicts [11]. Claire points out that the relationship between citizens and the government, their dependence on the region and their role in the decision-making mechanism all have an important impact on social acceptance research and the construction of NIMBY projects. And he has constructive ideas for improving effective citizen participation [12]. Therefore, improving public participation can effectively reduce the occurrence of NIMBY conflicts. In addition to the theoretical analysis of the NIMBY problem, the evolution process of NIMBY conflict can also be investigated from the perspective of mathematical modeling through the three-party game analysis. Zhang analyzed and summarized the characteristics of the waste management project, defined the core stakeholders, and described the unequal game process and results between the government and different stakeholders with damage based on game theory. And from the characteristics of the project, system and other reasons to analyze the causes of the unequal game, hope to strengthen the attention and help to the weak damaged stakeholders in the project [13]. Zhang comprehensively used game theory, social conflict analysis theory, stakeholder theory and risk theory to analyze the social conflict and NIMby phenomenon in the site selection, operation and management of waste disposal sites [14]. Liou and Chen to build the building group of environmental pollution emergency collaborative evolutionary game model, analyzed the environmental pollution incidents in the negotiations and black-box different disposal mode, and the different income and information situation caused by all kinds of co-evolution results, reveals the evolution of new phenomenon and law [15]. Chen and Lu et al. based on the theory of evolutionary game theory, the introduction of gaussian white noise random disturbance, build risk accumulation class construction enterprise in adjacent to avoid conflict with surrounding people two groups random evolutionary game model, comparison and analysis on the government and the government supervision group strategy choice behavior under the stochastic evolution process [16]. Yang from the perspective of scale politics in the region between the local government financial interest in the adjacent from the project location and adjacent to avoid events evolution process, and describes the adjacent event related government as well as the scale of the governments at higher levels of subject, game strategy and power relations change, at the same time analyses the scale reconstruction and conflict resolution mechanism [17]. Yi and Yang took three major stakeholders in the event——local governments, investors of dangerous goods logistics facilities and surrounding public as the research object to solve the conflict of site selection of dangerous goods logistics at home and abroad, and used evolutionary game theory to explore the influence of different interest appeals on the strategic choice of the three parties [18]. Based on stakeholder theory, Yu and Zhang constructed a three-party evolutionary game model of local government, new media and local people, and analyzed the interest relationship and role between local government, new media and local people in NIMBY conflict, which is of great significance, and deepened the understanding of multiple interest conflicts and their resolution [19]. Song and Liou consider the traditional mass protests surrounding the project of heavy pressure for social stability in environmental pollution incidents, built around the masses and the spontaneous evolutionary game model of the enterprise, government regulation is discussed under the surrounding people and enterprises controlled evolutionary game process, and explained the enterprise sewage and mass protests around the cyclical fluctuations [20]. It will provide useful reference for the government to achieve high-quality development and ensure employment. Zhou and Xu Using improved KMRW reputation model analysis of adjacent to avoid project construct a by arbitrary for the prisoner’s delegation, learning, and the reputation of the government and the public participation, the overall design for the project introduction, environment evaluation, compensation agreement, project construction, project operation of public participation in five stages adjacent multi stage from the project decision-making is game model. On this basis, the possibility and corresponding conditions of positive sum game are systematically analyzed, and a strategy of guiding citizens to participate in positive sum game is put forward by the local government [21]. Xu and Han considered the influence of risk preference factors on decision-making behaviors of subjects, introduced preference function to describe the risk attribute characteristics of subjects, constructed rank-dependent expected utility game model of NIMBY conflict, and analyzed the existence and characteristics of game equilibrium under different risk preferences of the government and the public [22]. Xu and Hu introduced the incentive coefficient to improve the traditional replication dynamic equation, and white Gaussian noise was introduced to describe the random disturbance during the evolution of NIMBY events, and constructed a stochastic evolution game model of NIMBY events under uncertain environment [23]. Jin and Wang introduced the signal game model to understand how information asymmetry affects the decision-making of the public and incineration plants in the construction process of waste incineration facilities. It provides a theoretical basis for studying the information asymmetry effect of NIMBY conflict and potential mitigation measures [24].

To sum up, the history and status quo of studies on NIMBY facilities at home and abroad are sorted out, the current situation of reconstruction of polluting NIMBY facilities enterprises is analyzed, and the relevant theories of NIMBY effect are sorted out. Through the evolutionary game theory, the behavior evolution process of reconstruction enterprises, government and public stakeholders in the reconstruction of NIMBY facilities is analyzed. At the same time, numerical simulation based on Matlab simulation platform is carried out to prove the effectiveness of the evolutionary theoretical model, which has certain theoretical and practical significance for the government and related departments to alleviate NIMBY conflicts.

## 3. Methodology

**Fig 1:**
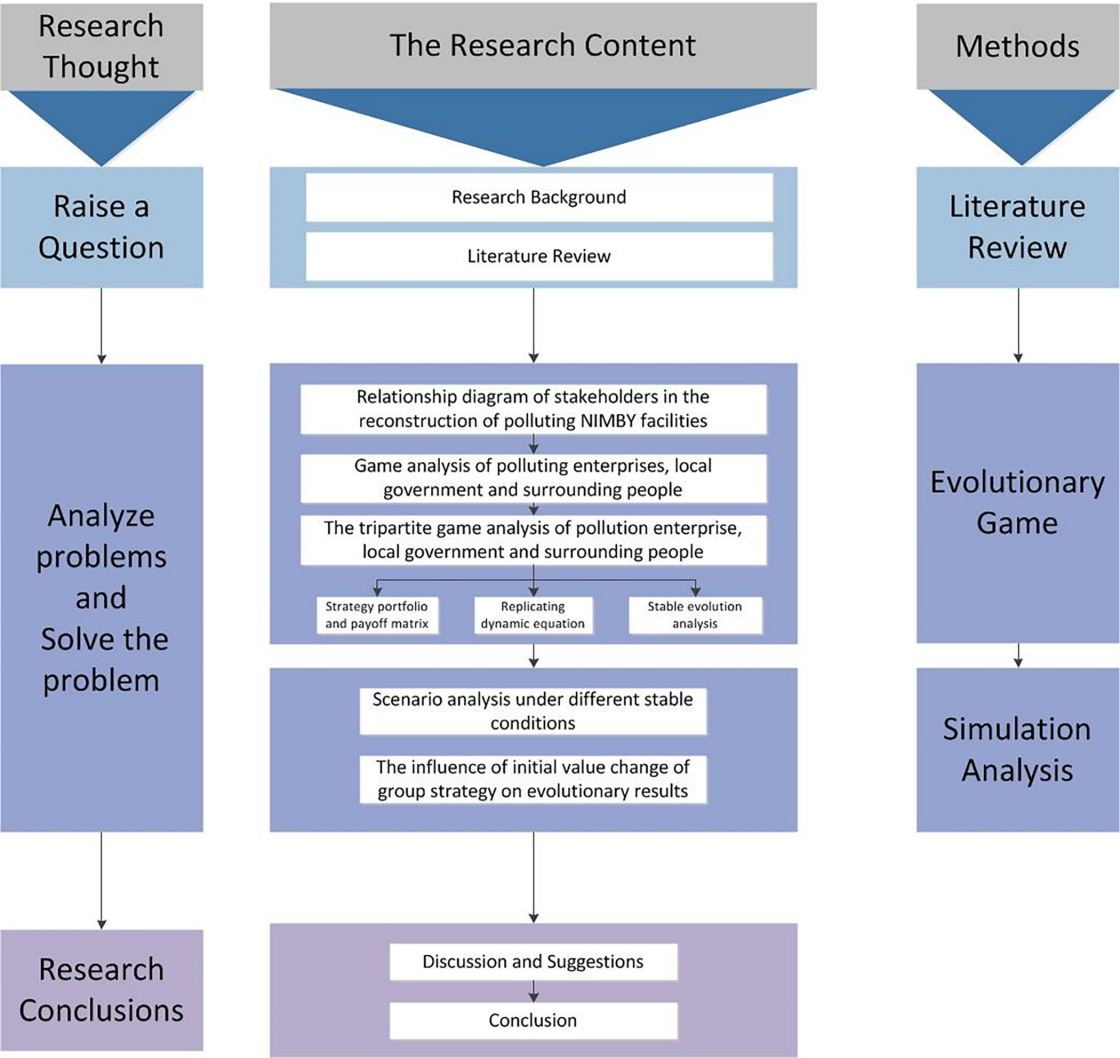
Method logic diagram.

Firstly, this paper sorted out the existing literature of NIMBY facilities through lit-erature research. By combing the literature and summarizing the existing research results of evolutionary game theory, stakeholder theory, environmental group event theory and polluting NIMBY facilities, the paper lays a theoretical foundation for further research on the evolutionary game problem of the reconstruction of polluting NIMBY facilities based on public participation behavior. Secondly, using the evolutionary game theory, assuming that the players in the game are bounded rational, enterprises, government and the public are regarded as the players in the game, seeking the optimal solution of the game, reaching the game equilibrium, and obtaining the stable strategy of the game model. Finally, by setting virtual parameters, MATLAB software is used to conduct numerical simulation analysis to verify whether the model evolves in accordance with the stability of the analysis. The logic diagram of the method of this paper is as following Fig 1.

### 3.1. Stakeholders of the reconstruction of polluting NIMBY facilities

**Fig 2:**
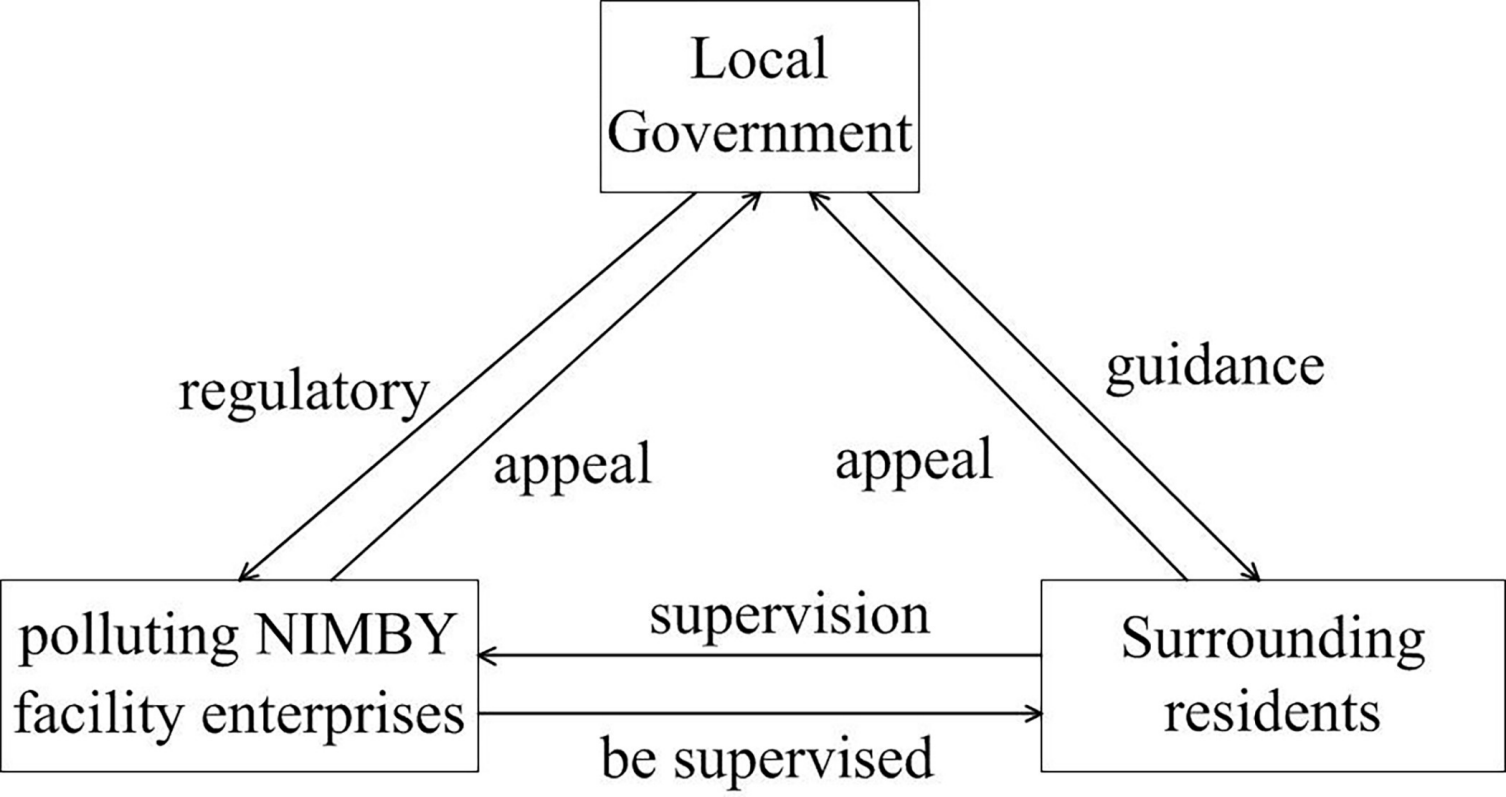
Stakeholders in NIMBY conflicts and their action mechanism.

The term stakeholder was originally used in enterprises. It was originally proposed by Freeman [25] in his book Strategic Management: An Analytical Approach to Stakeholder Management. Freeman believes that "stakeholders are individuals or groups that can influence the realization of organizational goals or are influenced by organiza-tional goals". This definition explains stakeholders in a broad sense, breaks through the aforementioned narrow definition and expands the content of stakeholders. Based on the stakeholder theory and the characteristics of NIMBY conflicts, this paper defines stakeholders in the reconstruction of polluting NIMBY facilities as follows: stakeholders in NIMBY conflicts refer to all individuals and groups that can influence the occurrence of NIMBY conflicts and are also affected by NIMBY conflicts. According to the genealogical theory, the change of interest of either party will cause the change of the mechanism of NIMBY conflict. Therefore, clarifying the interest relationship between the subjects of NIMBY conflict and realizing the balance of the interests of all parties play a crucial role in the resolution of NIMBY conflict. In order to simplify the study, the government, polluting NIMBY facility enterprises and the surrounding public are selected as the main stakeholders of NIMBY conflict Fig 2, and the role and mechanism played by the three stakeholders in the evolution of NIMBY conflict are systematically investigated.

### 3.2. The game relationship among stakeholders in the reconstruction of polluting NIMBY facilities

#### 3.2.1. The game between polluting NIMBY facilities enterprises and the government

In the early stages of urban development, NIMBY public facilities were built-in the outskirts of cities, far from residential areas, and the conflicts between local governments and polluting enterprises were not deep. But in recent years, the rapid development of cities, the expansion of urbanization area, earlier type adjacent to avoid public facilities have been built gradually surrounded by residential or commercial service, enterprise avoid polluting adjacent facilities near the harmful emissions of waste water, the health of the residents in the local government under the double pressure of the public and media, the interests of the relationship between enterprises have to reconsider and pollution. On the one hand, if the local government strictly regulates the polluting enterprises, it will need to invest a lot of time and supervision costs, but it will also bring potential benefits such as improving the image of the local government, and at the same time, the operating profit of the polluting enterprises will decrease. In this case, the polluting enterprises will adjust their strategies according to the punishment intensity of the local government. On the other hand, if local governments do not strictly regulate polluting enterprises, they can make more profits for polluting enterprises, but it will have a negative impact on their personal image and aggravate environmental pollution.

#### 3.2.2. The game between polluting NIMBY facilities enterprises and surrounding residents

In reconstruction of polluting NIMBY facilities, both the polluting NIMBY facilities enterprises and the public pursue the maximization of their own interests. Pollute NIMBY facility enterprises are linked to the immediate interests of the surrounding people, such as housing, and more importantly, to the threat to the living environment of the people. When the government carries out strict supervision and the fine cast is high, the polluting enterprises tend to adopt the way of environmental protection reconstruction, which may reduce the profit of the enterprises, but can alleviate the pollution to the surrounding situation. At this time, the polluting enterprises and the public form a good mutually beneficial relationship. However, when the government does not carry out meticulous supervision or carries out strict supervision but the penalty cost is low, the polluting enterprises prefer to choose the way of not carrying out reconstruction, so as to maximize their profits. At this time, the public will comprehensively consider the supervision cost and the potential benefits brought by a suitable living environment, and restrain the behavior of polluting enterprises through reporting and petitioning.

#### 3.2.3. The game between the local government and the surrounding people

In recent years, regional economic growth rate is among the important assessment criteria in the promotion mechanism of local officials. In this context, provincial officials will selectively regulate polluting enterprises to promote the economic growth of their region, so as to get the opportunity of rapid promotion. However, with the expansion of urban areas, previously built NIMBY public facilities are gradually surrounded by resi-dential areas or commercial service areas, which affect the living environment quality of surrounding residents. People around express their interest demands on the government by means of resistance, and the government alleviates the NIMBY conflict by strength-ening the supervision of polluting enterprises to reduce environmental pollution.

### 3.3. Establishment of evolutionary game model of polluting NIMBY facility reconstruction

#### 3.3.1. Basic assumption

Classical game theory requires players to be totally rational, which is an unrealistic assumption in the real world [26]. Evolutionary game theory assumes that participants in the game are bounded rational. Their decisions are realized by learning and imitating each other, and their strategies are constantly adjusted to reach an equilibrium state [27]. In the process of the game, local government, polluting NIMBY facilities enterprises and the public cannot find the optimal strategy at the very beginning. Instead, they gradually change their strategies in the process of learning and emulating the experience of their peers, and finally find the optimal strategy to take full advantage of their interests. The basic assumptions of this paper are as following:

Hypothesis 1: Without considering other constraints, polluting enterprises, government and surrounding people constitute a complete system, and all three are bounded rational individuals with learning ability.

Hypothesis 2: Because when the polluting NIMBY facilities enterprises were under construction, the surrounding environment was relatively empty and sparsely populated. Therefore, after the completion of residential or commercial areas, the enterprise can adopt two strategies: environmental protection reconstruction measures or not. For the environmental protection reconstruction of polluting NIMBY facilities, the public can choose two strategies of participating in supervision or not participating in supervision. Provincial governments can adopt two strategies: strict supervision or not strict supervision. In the process of multi-participants’ game, if the proportion of polluting NIMBY facilities enterprises adopting environmental protection reconstruction measures as *x*, then the proportion of non-adopting environmental protection reconstruction measures is 1−*x*, *x*∈[0, 1]. If the proportion of local government adopting strict supervision strategy is *y*, then the proportion of local government adopting non-strict supervision strategy is 1−*y*, *y*∈[0, 1]; If the proportion of the public adopting the strategy of participating in supervision is *z*, then the proportion of the public adopting the strategy of non-participating in supervision is 1−*z*, *z*∈[0, 1].

Hypothesis 3: In the evolutionary game system of dyeing NIMBY facility reconstruction, the participation of the government will affect the behavior decisions of the public and polluting enterprises. The participation behavior of the public and polluting enterprises depends on the reward and punishment mechanism designed by the government, and the purpose of supervising enterprise behavior is achieved by mobilizing the number of participants [28]. When the government chooses the strict supervision strategy, the government will give economic encouragement and support to the enterprises carrying out environmental protection reconstruction, so as to alleviate the marginal cost of environmental protection investment and make up for the cost of compliance, thus encouraging enterprises to increase investment in environmental protection [29]. At the same time, the government will also impose certain penalties on polluting enterprises that do not carry out environmental reconstruction. The combination effect of fines and subsidies can increase the promoting effect of environmental regulation on environmental investment [30]. For the surrounding people, the more the government rewards participants, the more the public will participate. The essence is to mobilize more public participation in supervision under this incentive and constraint framework, that is, to promote the transformation of individual behavior of public participation into group behavior, so as to supervise the environmental protection reconstruction behavior of polluting enterprises [28].

#### 3.3.2. Game payoff matrix

According to the above assumptions and analysis, a three-way evolutionary game model of polluting enterprises, the government and surrounding people in the operation, supervision and governance of polluting NIMBY facility enterprises can be constructed. Table 1 shows the meaning of the set parameters, and Table 2 shows the payoff matrix under the corresponding strategy.

**Table 1:** Parameter symbol and expression meaning.

symbol	Meaning of expression
W	Polluting enterprises in the normal circumstances of their earnings
C_l_	The cost incurred by polluting enterprises to purchase purification equipment and environmental protection materials
B	Polluting enterprises can be rewarded by local governments for making environmentally friendly improvements
L_1_	Potential losses to polluters due to mass resistance
L_2_	The potential loss to the public due to environmental pollution
S	Polluters are fined by the government when they fail to carry out environmental improvements under government and public supervision
C_g_	The cost to local governments of choosing strict monitoring strategies
R	After alleviating NIMBY conflicts, it will bring potential social benefits to local governments
F	Due to the aggravation of NIMBY conflicts, local governments will suffer from the negative benefits brought by the punishment of higher governments and the decline of credibility
C_p_	Costs incurred by the public for reporting polluters
e	Local government awards after public participation in supervision
P	Long-term benefits from the good living environment formed after the resolution of NIMBY conflicts

**Table 2:** Game payoff matrix.

Revenue matrix of tripartite game for operation supervision of polluting NIMBY facilities		The government			
		Strict supervision(y)		Lax regulation(1−y)	
		Polluting NIMBY facility enterprises		Polluting NIMBY facility enterprises	
		Adopt environmental protection reconstruction measures (x)	No environmental protection improvement measures will be taken (1−x)	Adopt environmental protection reconstruction measures (x)	No environmental protection improvement measures will be taken (1−x)
**The public**	Participate in supervision (z)	W−*C*_*l*_+B R−*C*_*g*_−e−B P+e−*C*_*p*_	W−*L*_1_−S S−*C*_*g*_−e−F e+P−*L*_2_−*C*_*p*_	W−C_l_ 0 P−C_p_	W−*L*_1_−S S−F −C_p_−*L*_2_
	Non-participation in supervision (1−z)	W−C_l_+B −Cg−B−F 0	W−*L*_1_−S S−C_g_−F −*L*_2_	W−C_l_ −F 0	W−*L*_1_ −F −*L*_2_

## 4. Game equilibrium analysis and simulation on the evolution of pollute NIMBY facilities reconstruction

### 4.1. Expected return function

#### 4.1.1. Expected revenue function of pollution-type NIMBY facility enterprises

Based on the evolutionary game model established above, the expected revenue of polluting NIMBY facility enterprises under different strategies can be obtained:

(1)Expected benefits of adopting environmentally friendly renovation technology for polluting NIMBY facilities enterprises are as follows:


$$U_{11}=yz(W-C_{l}+B)+y(1-z)(W-C_{l}+B)+(1-y)z(W-C_{l})+(1-y)(1-z)(W-C_{l})$$

(2)Expected benefits of polluting NIMBY facilities enterprises that do not adopt environmentally friendly renovation technologies are as follows:


$$U_{12}=yz(W-L_{1}-S)+y(1-z)(W-L_{1}-S)+(1-y)z(W-L_{1}-S)+(1-y)(1-z)(W-L_{1})$$

(3) The average expected earnings of polluting NIMBY facilities enterprises are as follows:


$$\bar{U_{1}}=xU_{11}+(1-x)U_{12}$$

#### 4.1.2. The expected revenue function of local government

Similarly, the expected revenue of local governments under different strategies can be obtained.

(1) The expected benefits of strict supervision by local governments are as follows:


$$U_{21}=xz(R-C_{g}-e-B)+x(1-z)(-C_{g}-B-F)+(1-x)z(S-C_{g}-e-F)+(1-x)(1-z)(S-C_{g}-F)$$

(2) The expected benefits of local governments that do not adopt strict supervision are:


$$U_{22}=xz\cdot 0+x(1-z)(-F)+(1-x)z(S-F)+(1-x)(1-z)(-F)$$

(3) The average expected revenue of local governments is:


$$\bar{U_{2}}=yU_{21}+(1-y)U_{22}$$

#### 4.1.3. The public’s expected return function

Similarly, the expected benefits of the public under different strategies can be obtained:

(1) The expected benefits of active public participation in supervision are as follows:


$$U_{31}=xy(P+e-C_{p})+(1-x)y(P+e-L_{2}-C_{p})+x(1-y)(P-C_{p})+(1-x)(1-y)(-C_{p}-L_{2})$$

(2) The expected benefits of not active public participation in supervision are as follows:


$$U_{32}=xy\cdot 0+(1-x)y(-L_{2})+x(1-y)\cdot 0+(1-x)(1-y)(-L_{2})$$

(3) The average expected benefit of the public is as follows:


$$\bar{U_{3}}=zU_{31}+(1-z)U_{32}$$

### 4.2. Replicative dynamic equations of games

Since the public, local government and polluting NIMBY facility enterprises and other players of the game mostly show bounded rationality, as time passes, the three parties of the game constantly adjust their strategic choices through learning and imitation, so as to make the best strategic decisions. When local governments, polluting NIMBY facilities enterprises and people around them adjust their strategic choices dynamically, they show the replicating dynamic process described by the evolutionary game theory, so the replicating dynamic equation is used to describe their evolution process [31]. The essence of replication dynamics is the dynamic differential equation of how often a particular strategy combination is adopted in a series of strategy combinations [32]. If a certain player of the game adopts a certain strategy to gain more than the average gain, the strategy can be developed. According to Table 2, the dynamic replication equation of the three-party game between the government, the polluting NIMBY facility enterprises and the neighboring people is as follows.

#### 4.2.1. Pollute NIMBY facilities replicate the dynamic equation

The replication dynamic equation of polluting NIMBY facility enterprises is as follows:

$$F(x)=\frac{dx}{dt}=(1-x)[y(B+S)+zS-yzS+L_{1}-C_{l}]$$

1. If $z=\frac{C_{l}-L_{1}-y(B+S)}{S-yS}$, then *F*(*x*) ≡ 0, which means that all horizontal states in this state are stable states, that is, the optimal strategy choice of the polluting NIMBY facility enterprise is to adopt the reconstruction strategy or not.
2. Indicates if the $z\neq \frac{C_{l}-L_{1}-y(B+S)}{S-yS},\;F(x)=0$, can get two stable point to: $x_{1}^{*}=0,\;x_{2}^{*}=1$. The derivative of *F*(*x*) gives that $F\prime (x)=(1-2x)[y(B+S)+zS-yzS+L_{1}-C_{l}]$

①When $z<\frac{C_{l}-L_{1}-y(B+S)}{S-yS}$, $F\prime (x){|}_{x_{1}^{*}=0}<0,\;F\prime (x){|}_{x_{2}^{*}=1}>0$. Thus, it can be seen that $x_{1}^{*}=0$ is the stable point, at which polluting NIMBY facilities enterprises choose not to take environmental protection reconstruction measures.

②When $z>\frac{C_{l}-L_{1}-y(B+S)}{S-yS}$, $F\prime (x){|}_{x_{1}^{*}=0}>0$, $F\prime (x){|}_{x_{2}^{*}=1}<0$. Thus, it can be seen that $x_{2}^{*}=1$ is the stable point, at which polluting NIMBY facilities enterprises choose to adopt environmental protection reconstruction measures.

#### 4.2.2. Local governments replicate dynamic equations

The replication dynamic equation of local government is:

$$G(y)=\frac{dy}{dt}=y(1-y)[x(-B-S)+z(-e-S)+xz(R+S)+S-C_{g}]$$

1. If $x=\frac{\left(C_{g}-S)+z(e+S\right)}{z(R+S)-(B+S)}$, then *G*(*y*) ≡ 0, which means that all horizontal states in this state are stable states, that is, the local government adopts strict supervision or does not adopt strict supervision is its optimal strategy choice.
2. If $x\neq \frac{\left(C_{g}-S)+z(e+S\right)}{z(R+S)-(B+S)}$, then *y* can be obtained two stable point to: $y_{1}^{*}=0,\;y_{2}^{*}=1$. The derivation of *G*(*y*) can get $G\prime (y)=(1-2y)[x(-B-S)+z(-e-S)+xz(R+S)+S-C_{g}]$

①When $x<\frac{\left(C_{g}-S)+z(e+S\right)}{z(R+S)-(B+S)},\;G\prime (y){|}_{y_{1}^{*}=1}<0,\;G\prime (y){|}_{y_{2}^{*}=1}>0$, then $y_{1}^{*}=0$ is the stable point, chose not to tight regulation strategy of local government at this time.

②When $x>\frac{\left(C_{g}-S)+z(e+S\right)}{z(R+S)-(B+S)},\;G\prime (y){|}_{y_{1}^{*}=0}>0,\;G\prime (y){|}_{y_{2}^{*}=1}<0$, the $y_{2}^{*}=1$ is a stable point, the local government regulation strategy choice.

#### 4.2.3. The public replicates dynamic equations

The public replication dynamic equation is:

$$H(z)=\frac{dz}{dt}=z(1-z)[xP+y(P+e)+xy(-P)-C_{p}]$$

1. If $y=\frac{C_{p}-xP}{(1-x)P+e}$, then *H*(*z*) ≡ 0, which means that all horizontal states in this state are stable states, that is, the public to take supervision or not to take supervision strategy is its optimal strategy choice.
2. If $y\neq \frac{C_{p}-xP}{(1-x)P+e}$, then *H*(*z*) = 0, can get z two stable point to: $z_{1}^{*}=0,\;z_{2}^{*}=1$. The derivative of *H*(*z*) tells us that $H\prime (z)=(1-2z)[ye-C_{p}]$.

①When $y<\frac{C_{p}-xP}{(1-x)P+e},\;H\prime (z){|}_{z_{1}^{*}=0}<0,\;H\prime (z){|}_{z_{2}^{*}=1}>0$, then $z_{1}^{*}=0$ is the stable point, the public option not supervision measures.

②When $y>\frac{C_{p}-xP}{(1-x)P+e}$ when $H\prime (z){|}_{z_{1}^{*}=0}>0,\;H\prime (z){|}_{z_{2}^{*}=1}<0$, the $z_{2}^{*}=1$ is a stable point, the public option supervision measures.

### 4.3. Analysis on the evolutionary stability of the strategy of tripartite subject in game

By *F*(*x*), *G*(*y*), *H*(*z*) can be a enterprises avoid polluting adjacent facilities, local government and the public power system consisting of three parties(I)

$$\left\{ \begin{array}{c} F(x)=x(1-x)[y(B+S)+zS-yzS+L_{1}-C_{l}]\\ G(y)=y(1-y)[x(-B-S)+z(-e-S)+xz(R+S)+S-C_{g}]\\ H(z)=z(1-z)[xP+y(P+e)+xy(-P)-C_{p}] \end{array}\right.$$ (I)

In order to get the system balance, according to the Eq (I),

$$\left\{ \begin{array}{c} F(x)=0\\ G(y)=0\\ H(z)=0 \end{array}\right.$$

For the solution to eight special equilibrium: (0, 0) (0, 1) (0, 0) (0,1,1) (0, 1) (1, 1) (1, 0) (1,1,1).

According to Lyapunov stability theory, the asymptotic stability of the system can be judged by establishing the Jacobian matrix of the equation and analyzing the eigenvalues of the matrix at each equilibrium point. The Jacobi matrix Eq (I) *J* for:

$$J=\left|\begin{array}{ccc} \frac{\partial F(x)}{\partial x} & \frac{\partial F(x)}{\partial y} & \frac{\partial F(x)}{\partial z}\\ \frac{\partial G(y)}{\partial x} & \frac{\partial G(y)}{\partial y} & \frac{\partial G(y)}{\partial z}\\ \frac{\partial H(z)}{\partial x} & \frac{\partial H(z)}{\partial y} & \frac{\partial H(z)}{\partial z} \end{array}\right|=\left|\begin{array}{ccc} \left(1-2x)[y(B+S)+zS-yzS+L_{1}-C_{l}\right] & x(1-x)[(B+S)-zS] & x(1-x)(S-yS)\\ y(1-y)[z(R+S)-(B+S)] & \left(1-2y)[x(-B-S)+z(-e-S)+xz(R+S)+S-C_{g}\right] & y(1-y)[x(R+S)-(e+S)]\\ z(1-z)(1-y)P & z(1-z)[(1-x)P+e] & \left(1-2z)[xP+y(P+e)+xy(-P)-C_{p}\right] \end{array}\right|$$

Eight special equilibrium points were substituted into the Jacobian matrix respectively. Thus the eigenvalues of the corresponding Jacobian matrix were obtained. Table 3 shows the eigenvalues of each equilibrium point.

**Table 3:** Eigenvalues of each equilibrium point.

Equilibrium	The eigenvalue		
	λ_1_	λ_2_	λ_3_
(0,0,0)	−*C*_*l*_+*L*_1_	S−*C*_*g*_	−*C*_*p*_
(0,0,1)	−*C*_*l*_+*L*_1_+S	−*C*_*g*_−e	*C*_*p*_
(0,1,0)	−*C*_*l*_+B+*L*_1_+S	−S+*C*_*g*_	−*C*_*p*_+e+P
(0,1,1)	−*C*_*l*_+B+*L*_1_+S	*C*_*g*_+e	*C*_*p*_−e−P
(1,0,0)	−*L*_1_+*C*_*l*_	−B−*C*_*g*_	−*C*_*p*_+*P*
(1,0,1)	*C*_*l*_−*L*_1_−S	−B−*C*_*g*_+R−e	*C*_*p*_−*P*
(1,1,0)	*C*_*l*_−B−*L*_1_−S	B+*C*_*g*_	−*C*_*p*_+*e*+*P*
(1,1,1)	*C*_*l*_−B−*L*_1_−S	B+*C*_*g*_−R+e	*C*_*p*_−*e*−*P*

According to Table 3, (0,0,1) can only be the unstable point, because *λ*_3_ = *C*_*p*_>0 is always true; (0,1,1) can only be an unstable point, because *λ*_2_ = *C*_*g*_+e>0 is always true; (1,1,0) is also an unstable point, because *λ*_2_ = B+*C*_*g*_>0 is always true;In the case of Table 4 stability conditions, (0, 0) (0, 0) (0, 1) (1, 1) (1,1,1) may be ESS.

**Table 4:** Stability conditions of equilibrium points.

The balance	Stability condition	Serial number
(0,0,0)	−*C*_*l*_+*L*_1_<0 S−*C*_*g*_<0 −*C*_*p*_<0	①
(0,1,0)	−*C*_*l*_+B+*L*_1_+S<0 −S+*C*_*g*_<0 −*C*_*p*_+e+P<0	②
(1,0,0)	−*L*_1_+*C*_*l*_<0 −B−*C*_*g*_<0 −*C*_*p*_+*P*<0	③
(1,0,1)	*C*_*l*_−*L*_1_−S<0 −B−*C*_*g*_+R−e<0 *C*_*p*_−*P*<0	④
(1,1,1)	*C*_*l*_−B−*L*_1_−S<0 B+*C*_*g*_−R+e<0 *C*_*p*_−*e*−*P*<0	⑤

### 4.4 The simulation analysis

#### 4.4.1 Scenario analysis under different conditions

According to the stability analysis of the game model, the strategic choices of local government, polluting NIMBY facility enterprises and the public in the reconstruction of polluting NIMBY facilities are different under different stability conditions.为In this paper. Matlab is used to carry out a numerical simulation of the evolution process of strategy combination under the above three stability conditions.

**Scenario 1** The polluting NIMBY facilities enterprises, local governments and the public adopt (not adopt environmental protection reconstruction, not strictly supervise, not participate in the supervision) strategy evolution.

Set the parameters as *C*_*l*_ = 16, B = 3, *L*_1_ = 3, S = 5, *C*_*g*_ = 7, R = 9, *C*_*p*_ = 6, e = 2, P = 3.

**Fig 3:**
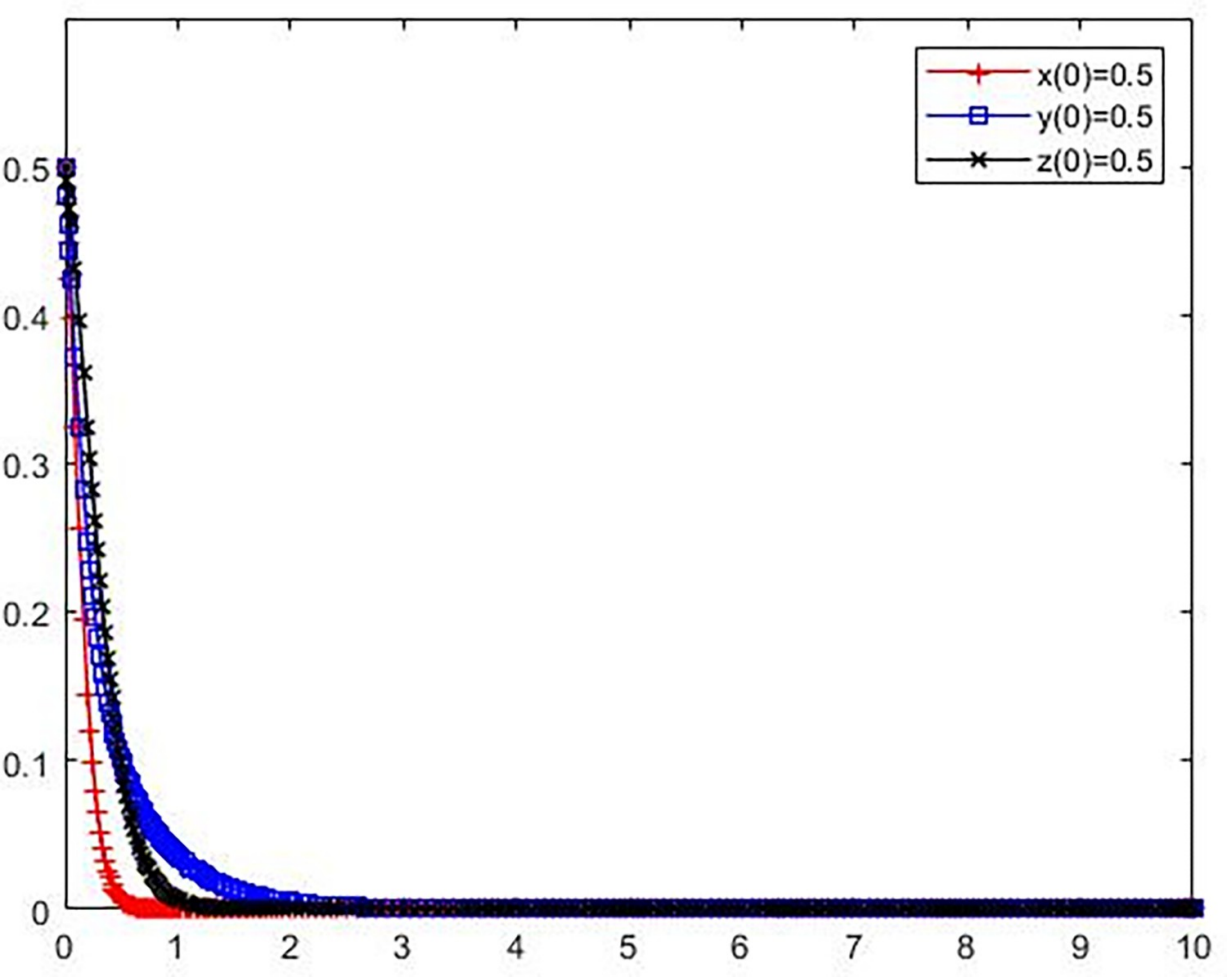
Evolution trajectory of game subject under stable condition ①.

The initial policy values of polluting NIMBY facilities enterprises, local governments and the public were set as x(0) = 0.5, y(0) = 0.5, z(0) = 0.5. The simulation results are presented in Fig 3.

It can be seen from Fig 1 that when the parameter values meet the stability condition ① −*C*_*l*_+*L*_1_<0, S−*C*_*g*_<0, −*C*_*p*_<0, That do not take environmental protection measures to avoid polluting adjacent facilities enterprises avoid the potential loss of less than polluting adjacent facilities enterprise environmental modification cost, polluting enterprises do not take environmental protection measures to avoid facilities of fines strict supervision, public supervision and costs less than local government is greater than zero, the tripartite game subject tends to take (do not take environmental reconstruction, strict supervision, to participate in supervision) strategy, at this point (0,0,0) is the ESS. This indicates that as long as the cost of strict supervision by local governments is too high or the amount of money for polluting NIMBY facilities enterprises is too low, local governments will choose the strategy of lax supervision if they have no profit in the supervision action. In addition, local governments’ relaxation of supervision will reduce the external threat and pressure of environmental protection reconstruction of polluting NIMBY facilities enterprises, and they will gain greater benefits if they do not adopt environmental protection reconstruction strategy. When local governments and polluting NIMBY facilities enterprises both adopt negative strategies, the public has to choose the non-supervision strategy because the cost of supervision is greater than the benefit. In this case, the players of the three-party game all adopt negative governance attitude, the system enters the worst state, and the NIMBY conflict becomes more serious.

**Scenario 2.** Pollution-oriented NIMBY facilities enterprises, local governments and the public adopt the strategic evolution (no environmental reconstruction, strict supervision, no participation in supervision).

**Fig 4:**
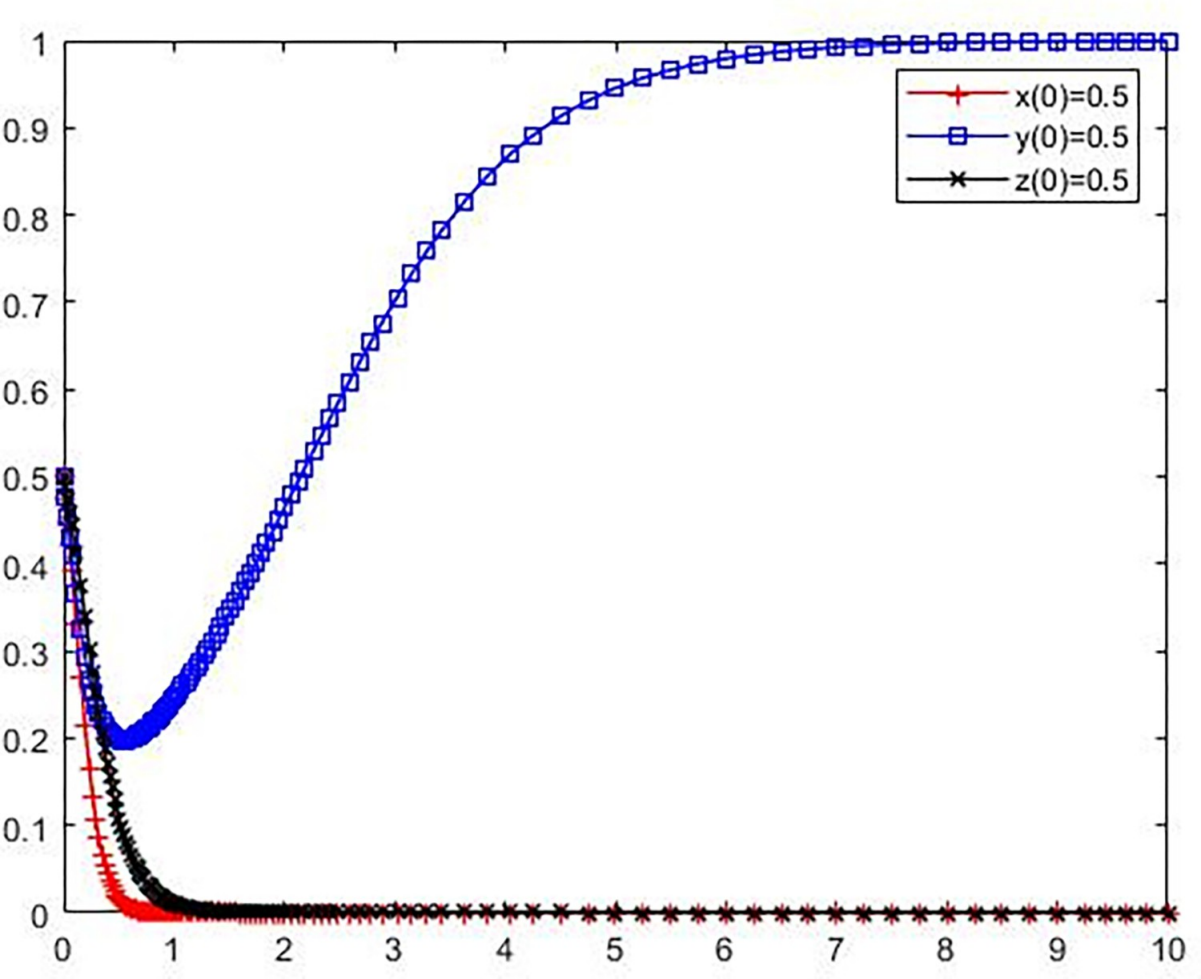
Evolution trajectory of game subject under stable condition ②.

Set the parameters as *C*_*l*_ = 16, B = 3, *L*_1_ = 3, S = 8, *C*_*g*_ = 7, R = 9, *C*_*p*_ = 6, e = 2, P = 3. Compared with Scenic 1, it only increases the value of the fine amount S for polluting NIMBY facilities enterprises that do not take environmental protection reconstruction measures. The initial policy values of polluting NIMBY facilities enterprises, local governments and the public were set as x(0) = 0.5, y(0) = 0.5, z(0) = 0.5. The simulation results are presented in Fig 4.

From Fig 4 shows that the parameter values satisfy the stability condition ② r*C*_*l*_+B+*L*_1_+S<0, −S+*C*_*g*_<0, and −*C*_*p*_+e+P<0, the polluting enterprises are not adjacent to avoid facility environmental reconstruction potential losses and fined the sum of less than avoid polluting adjacent facilities environmental renovation award and the difference between the cost of enterprise, strict supervision of local government is less than the cost of polluting enterprises are not adjacent to avoid facility environmental fines of the renovation, and public scrutiny of the long-term benefits and the government to the public the reward of the sum is less than the public supervision cost, equilibrium is ESS (0,0,0). For the local government, fines of polluting NIMBY facilities enterprises that do not take environmental protection reconstruction measures are the benefits under strict supervision. The increase of fines benefits the local government in the supervision action, and it will naturally choose the strict supervision strategy driven by the goal of profit maximization. Although the fines imposed on polluting NIMBY facilities enterprises for not taking environmental protection reconstruction measures will also have a certain impact on the strategic choice of polluting NIMBY facilities enterprises, the fines are not enough to force them to choose environmental protection reconstruction strategies relative to the difference between the cost of reconstruction and government subsidies.

**Scenario 3.** The polluting NIMBY facilities enterprises, local governments and the public adopt (adopt environmental protection reconstruction, do not strictly supervise, do not participate in the supervision) strategy evolution.

**Fig 5:**
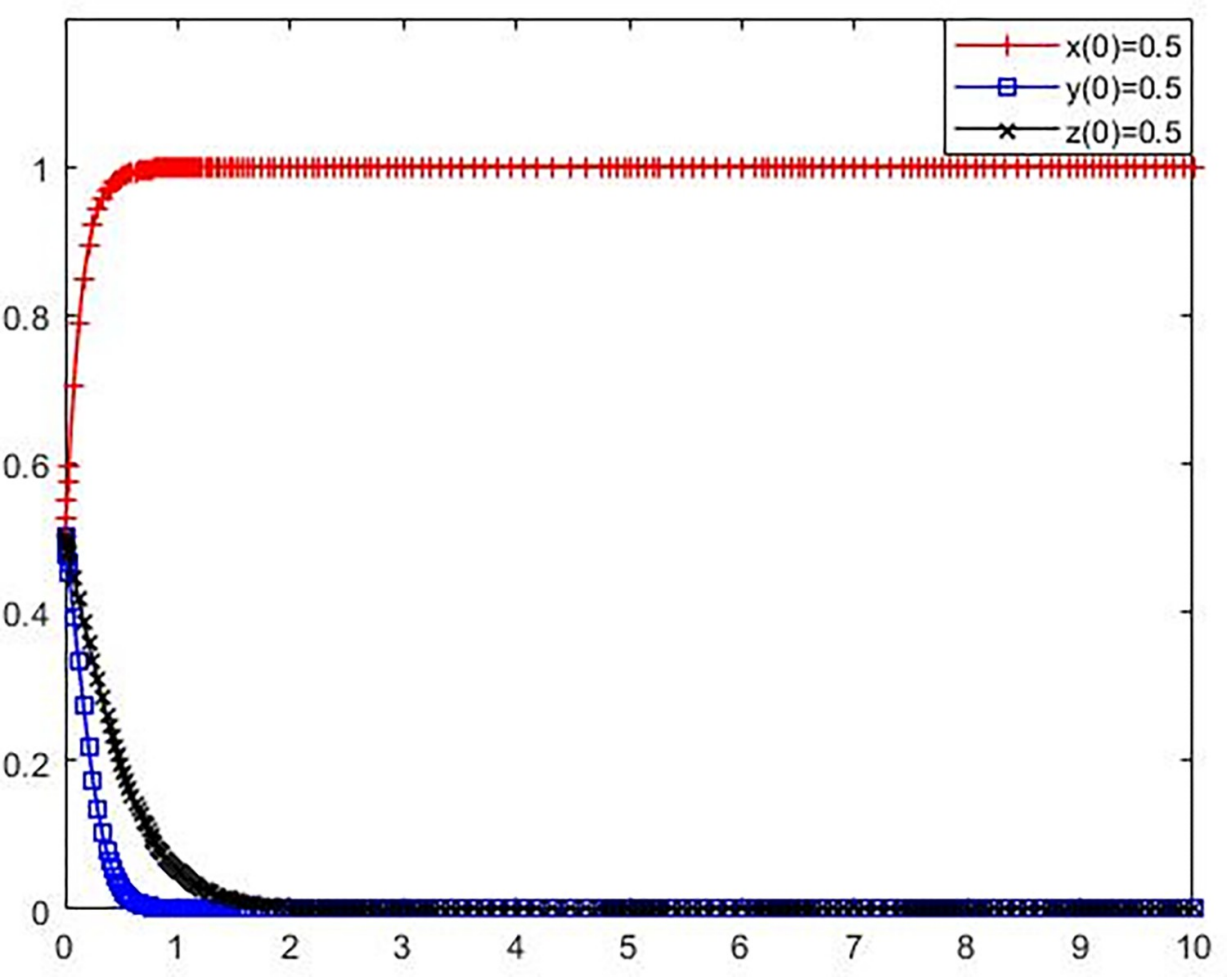
Evolution trajectory of game subject under stable condition ③.

Set the parameters as *C*_*l*_ = 16, B = 3, *L*_1_ = 20, S = 5, *C*_*g*_ = 7, R = 9, *C*_*p*_ = 6, e = 2, P = 3. Compared with scenario 1, the value of potential loss *L*_1_ brought by not taking environmental protection reconstruction measures to the polluting NIMBY facility enterprises is increased. The initial policy values of polluting NIMBY facilities enterprises, local governments and the public were set as x(0) = 0.5, y(0) = 0.5, z(0) = 0.5. The simulation results are shown in Fig 5.

From Fig 5 shows that the parameter values satisfy the stability condition ③ −*L*_1_+*C*_*l*_<0, −B−*C*_*g*_<0, and −*C*_*p*_+*P*<0, that is, avoid polluting adjacent facilities, environmental modification cost is less than the environmental modification not give enterprise to bring the potential loss of avoid polluting adjacent facilities, local governments to avoid polluting adjacent facilities enterprise environmental modification of reward and strict supervision cost the sum is greater than zero, the supervision from the public’s long-term benefits is less than the public supervision cost, equilibrium (1,0,0) is the ESS. This indicates that increasing the potential loss of polluting NIMBY facilities enterprises not carrying out environmental protection reconstruction is an important measure to force polluting NIMBY facilities enterprises to choose the way of environmental protection reconstruction. Such loss may lead to corporate image damage and market recognition decrease due to the NIMBY conflict between substandard emissions and surrounding residents. However, compared with the local government and the public, the potential loss of polluting NIMBY facilities enterprises not carrying out environmental protection reconstruction will not have a great impact on them, so they still maintain a negative supervision attitude.

**Scenario 4.** The strategic evolution of polluting NIMBY facilities enterprises, local governments and the public to adopt (adopt environmental protection reconstruction, not strictly supervise, participate in supervision).

**Fig 6:**
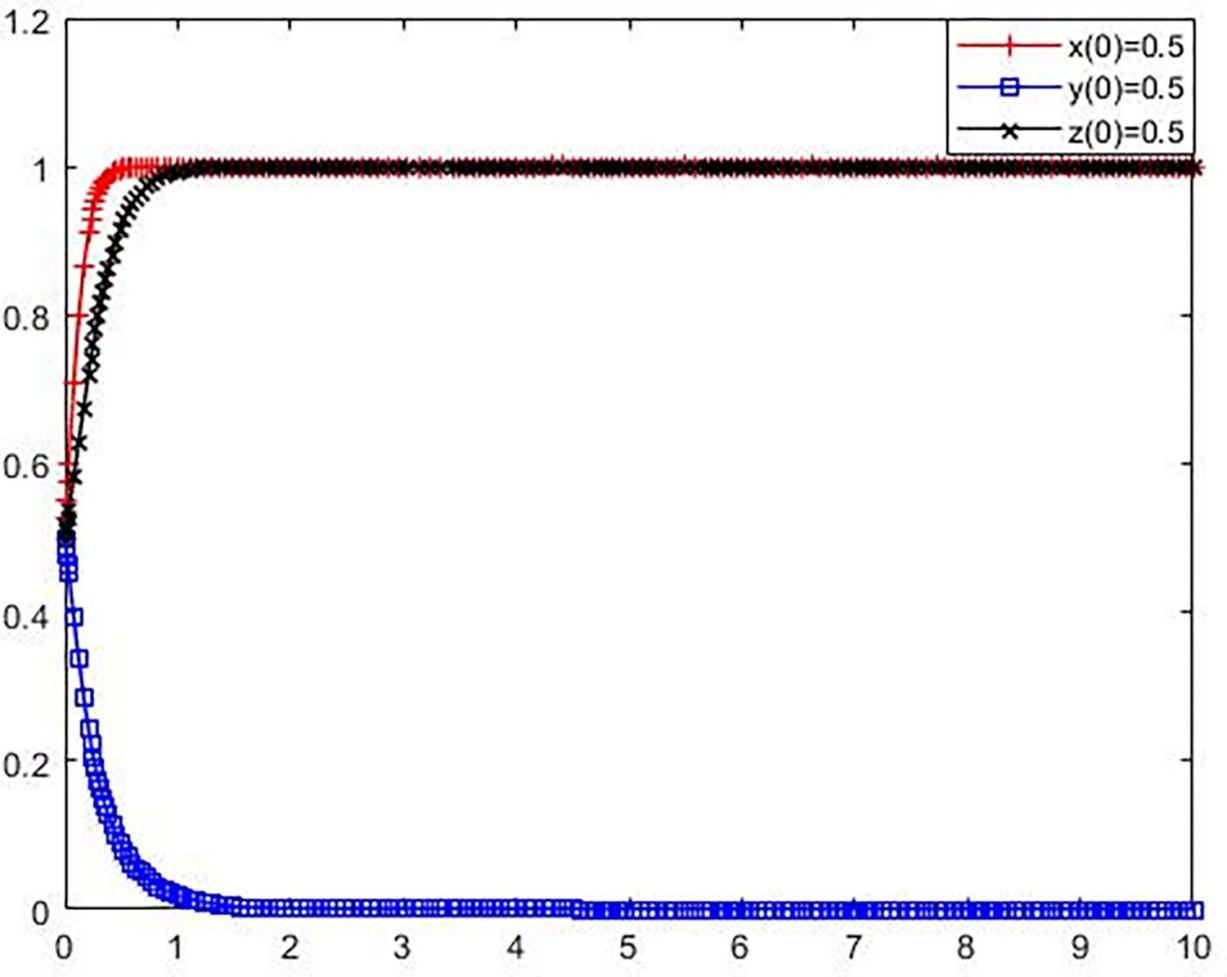
Evolution trajectory of game subject under stable condition ④.

Set the parameters as *C*_*l*_ = 16, B = 3, *L*_1_ = 20, S = 5, *C*_*g*_ = 7, R = 9, *C*_*p*_ = 3, e = 2, P = 8. Compared with Scenario 3, it reduces the value of *C*_*p*_ of public supervision cost and add value to P of long-term benefits brought by public supervision. The initial policy values of polluting NIMBY facilities enterprises, local governments and the public were set as x(0) = 0.5, y(0) = 0.5, z(0) = 0.5. The simulation results are shown in Fig 6.

From Fig 6 shows that the parameters satisfy the stability condition④ *C*_*l*_−*L*_1_−S<0, −B−*C*_*g*_+Roe<0, and *C*_*p*_−*P*<0, that is, avoid polluting adjacent facilities enterprise environmental renovation costs less than the potential loss of not rebuilt and fined the sum of the, strict supervision of local government’s potential benefits is less than the local government for the costs of all other subject rewards and strict supervision, public supervision and cost less than the long-term earnings of supervision, equilibrium (1,0,1) is the ESS. This indicates that the public will comprehensively consider their own benefits and participation costs when making strategic choices, and increasing the benefits and reducing the costs can effectively promote public participation in supervision.

**Scenario 5.** Enterprises of polluting NIMBY facilities, local governments and the public adopt (adopt environmental protection reconstruction, strict supervision, participate in supervision) strategy evolution.

**Fig 7:**
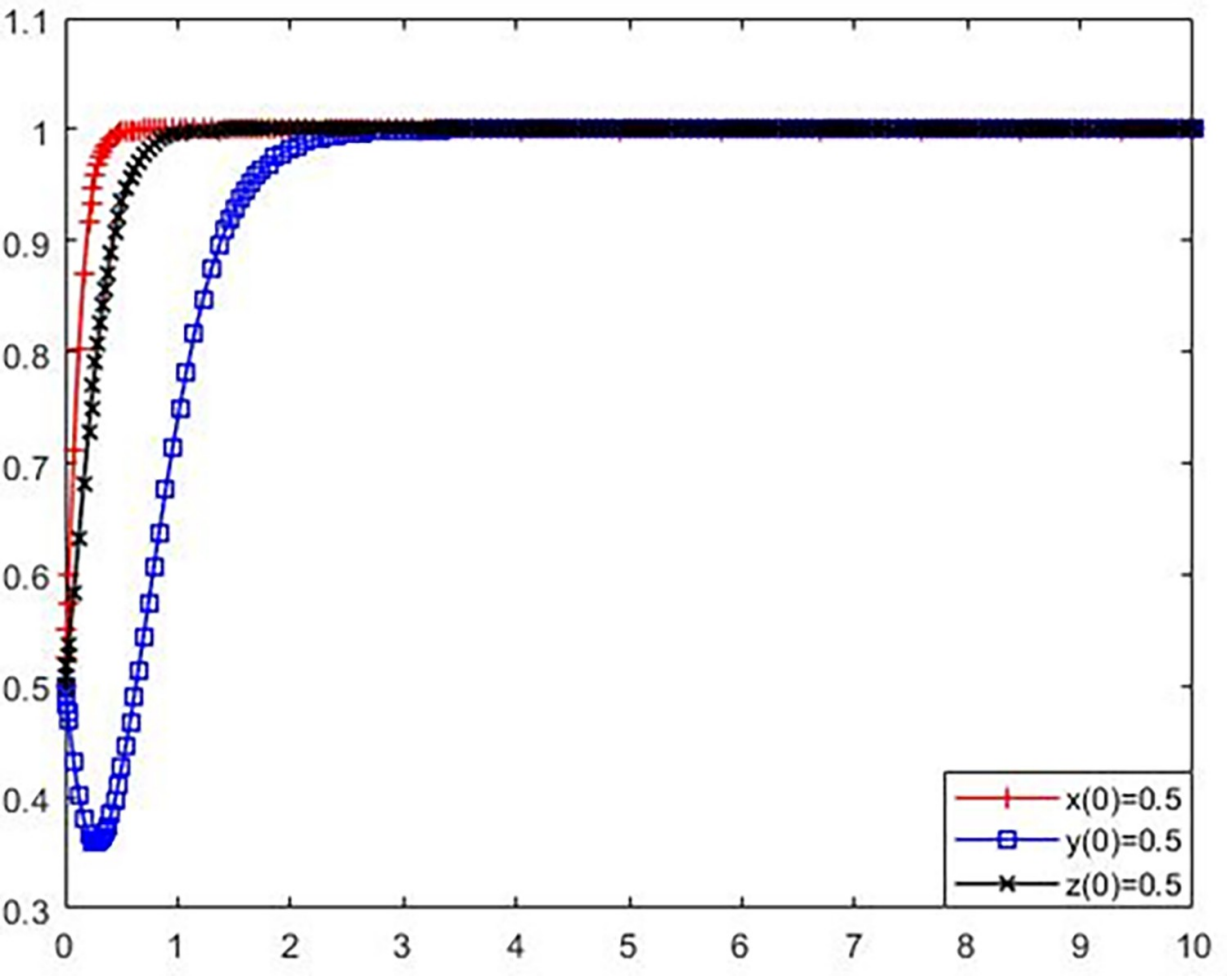
Evolution trajectory of game subject under stable condition ⑤.

Set the parameters as *C*_*l*_ = 16, B = 3, *L*_1_ = 20, S = 5, *C*_*g*_ = 7, R = 15, *C*_*p*_ = 3, e = 2, P = 8. Compared with Scenario 4, it only improves the value of R of the potential revenue when the local government strictly supervises. The initial policy values of polluting NIMBY facilities enterprises, local governments and the public were set as x(0) = 0.5, y(0) = 0.5, z(0) = 0.5. The simulation results are shown in Fig 7.

From Fig 7 shows that the parameter values satisfy the stability condition ⑤ *C*_*l*_−B−*L*_1_−S<0, B+*C*_*g*_−R+e<0, and *C*_*p*_−*e*−*P*<0, that is, avoid polluting adjacent facilities enterprise environmental renovation costs and rewards is less than the difference between the enterprise avoid polluting adjacent facilities not potential losses and fined the sum of the renovation, the local government to other subject rewards and strict governance cost the sum is less than the potential benefits of strict supervision of local government, and the public supervision and cost less than public oversight of the long-term benefits and rewards when the sum of the local government to the public supervision, equilibrium(1,1,1) is ESS. This indicates that when both polluting NIMBY facilities enterprises and the public are inclined to actively participate in environmental protection reconstruction and supervision, the system can enter a virtuous cycle as long as the potential benefits obtained by the local government in the supervision, such as the enhancement of public image and credibility.

#### 4.4.2 The influence of initial value change of group strategy on evolutionary results

According to analysis of the replication dynamic equation, the evolutionary equilibrium state of the players in one game is affected by the decision-making proportion of other players. Considering the singularity of the initial value of the main strategy of the tripartite game in the above scenario analysis, this paper tests the evolution results of group strategy by adjusting the initial value, so as to eliminate the influence of the initial value selection on the credibility of the results and enhance the persuasion of the results.

**Fig 8:**
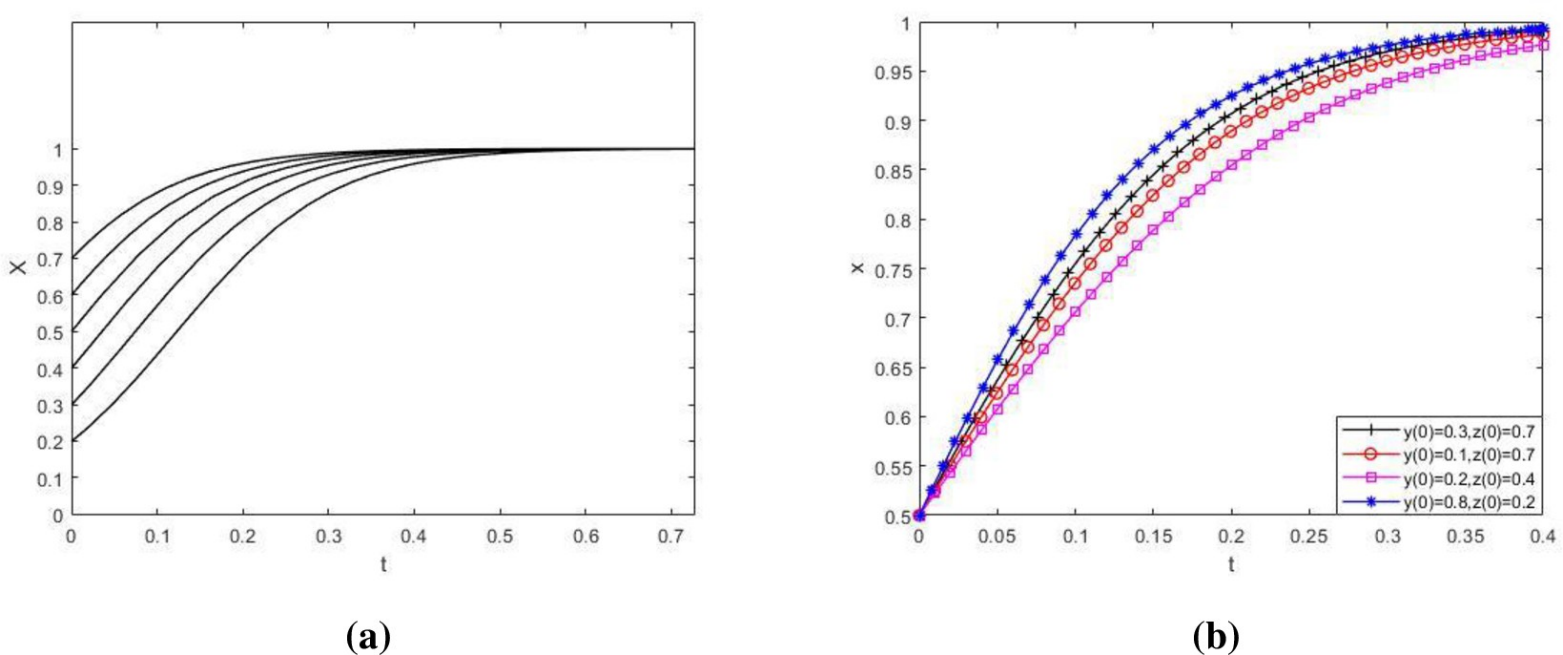
The influence of initial value change on the strategy evolution path of NIMBY enterprises. Influence of initial value change of X on strategy evolution of NIMBY enterprises; (b) Influence of initial value change of Y and Z on strategy evolution of NIMBY enterprises. Fig 9 to investigate the influence of the change of initial value of the main strategy of the tripartite game on the evolutionary path of local government’s group strategy. As showed in the figure, Y value first decreases and then increases over time (Fig 9A and Fig 9B). This may be because at the beginning, NIMBY conflict is not obvious, just a small protest by nearby residents, and the cost of local government supervision is greater than the benefits gained. At this time, the local government does not pay attention to the phenomenon of NIMBY conflict, so it will not take supervisory action. Later, as time went on, the NIMBY conflict became more and more serious. Residents nearby protested in a large scale and even put pressure on the government through public opinion. If the government strictly supervises and solves the conflicts between polluting NIMBY facilities enterprises and nearby residents, the public image and credibility of the government will be greatly improved, far exceeding the cost of government supervision, which makes the local government adopt strict supervision strategy and eventually evolve to a balanced state.

**Fig 9:**
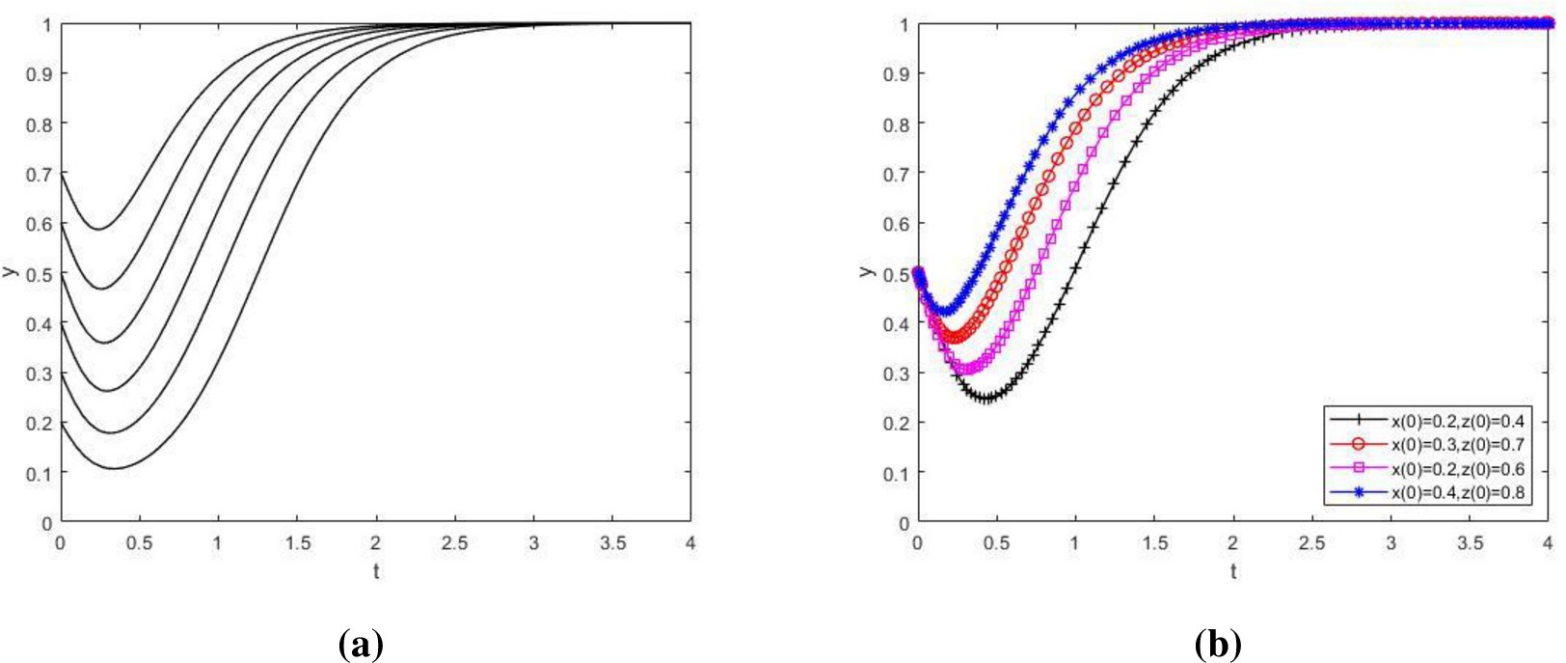
The influence of initial value change on the strategy evolution path of local government. (a) Influence of initial value change of Y on strategy evolution of local government; (b) Influence of initial value change of X and Z on strategy evolution of local government. Fig 10 to investigate the influence of the change of initial value of the main body’s strategy in the tripartite game on the evolutionary path of the public group’s strategy. As showed in the figure, although the evolution speed of public group strategy varies when the initial value of the main body strategy in the three-party game changes, the evolution result of z→1 does not change (Fig 10A and Fig 10B). The public has historically been playing the role of victim in NIMBY conflicts. The negative externalities generated by NIMBY facilities have an increasing impact on the production and life of the public. Therefore, the public will get a more positive attitude towards environmental protection reconstruction of polluting NIMBY facilities enterprises.

**Fig 10:**
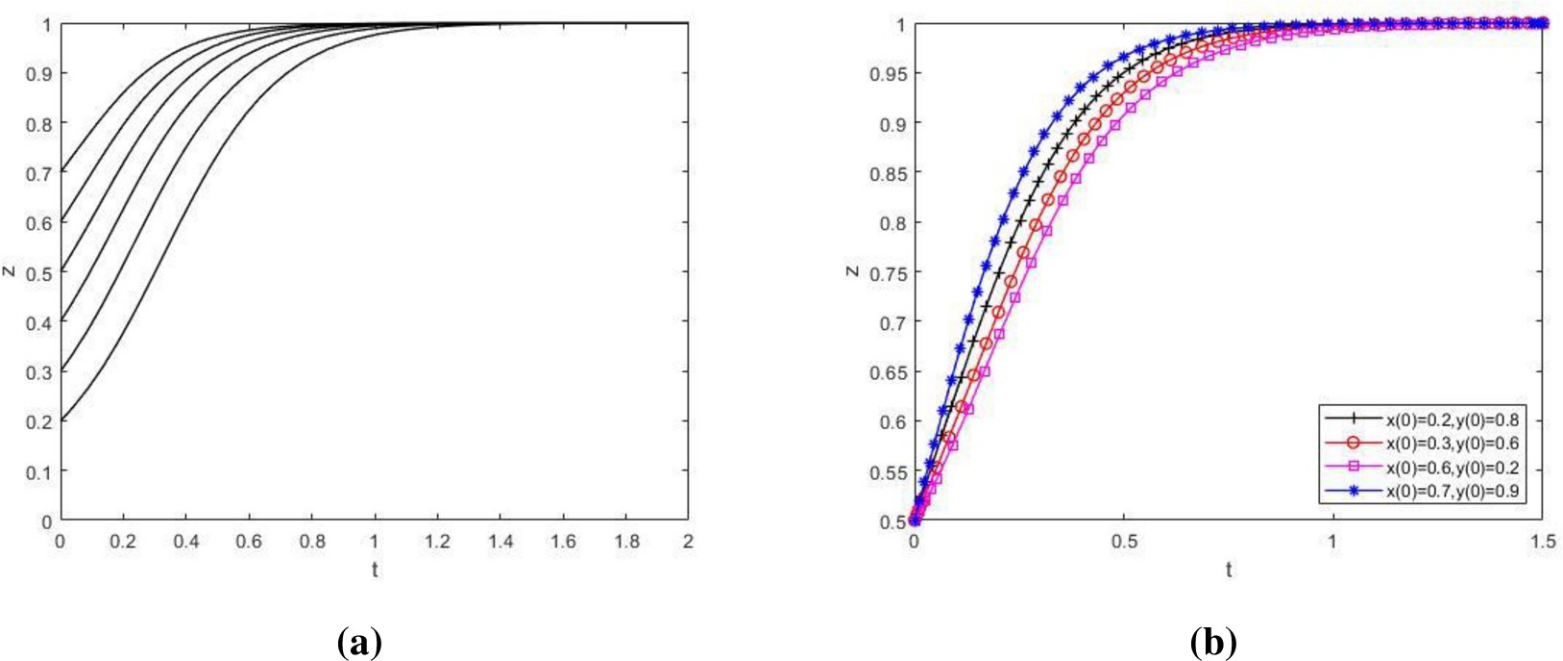
The influence of initial value change on public strategy evolution path. (a) Influence of initial value change of Z on public strategy evolution; (b) Influence of initial value change of X and Y on public strategy evolution.

Firstly, when the parameter value meets the stability condition ⑤, the influence of the change of the initial value of the main strategy of the tripartite game on the evolution result is studied, as showed in Figs 8–10. At this time, the parameter value is as that in Scenario 5.

Fig 8 investigates the influence of the change of initial value of the main strategy in the tripartite game on the evolutionary path of the group strategy of polluting NIMBY facility enterprises. As showed in the figure, the larger the proportion of polluting NIMBY facility enterprises choosing environmental protection reconstruction strategy is, the shorter and faster system evolvement time to x→1 will be (Fig 8A). In addition, the evolution speed of this group will also be affected by the change of initial value of local government and public strategy (Fig 8B). It shows that on the premise of satisfying the interest demands of all parties, although the differences in initial values of x, y and z will have a certain influence on the strategic evolution path of polluting NIMBY facility enterprises, they will not change the final strategic choice of this group.

In conclusion, when the parameter values meet the stability condition ⑤, the change of initial strategic values of polluting NIMBY facility enterprises, local governments and the public will affect the evolution speed of the players, but will not change the final strategic choice of the players in the game. In this case, the equilibrium point (1,1,1) is ESS.

Similarly, the above method was used to investigate the influence of initial value changes in the strategy of the evolution results under the other four stable conditions, and it was found that the evolutionary stability strategy would not change with the change of initial value selection. In other words, the results in scenario analysis were credible. In view of space limitation, the other four cases are not elaborated in detail in this paper.

#### 4.4.3 Pareto optimal solution

According to the parameter values of scenarios 1–5, and assume that W = 30, F = 10, *L*_2_ = 5. According to the income matrix in Table 2, the income of enterprises, government and the public is shown in Table 5.

**Table 5:** Tripartite benefits.

The balance	Tripartite benefits
(0,0,0)	(-10,27,-5)
(0,1,0)	(-9,19,-5)
(1,0,0)	(-10,14,0)
(1,0,1)	(0,14,5)
(1,1,1)	(3,17,7)

According to the Pareto efficiency criterion [33], economic efficiency is reflected in the allocation of social resources to improve people’s situation, especially depending on whether the resources have been fully utilized. If the resource is fully used, and I have to hurt you to improve it, or you have to hurt me to improve it, then an economic process is said to have achieved a Pareto efficiency optimum.

By shown in Table 5, in five kinds of situations in the enterprise, government, public benefits, respectively (10, 27, - 5), (9, 3–5), (- 10,14,0), (0,14,5), (3,17,7). It follows that only (1,1,1) is pareto optimal. At this point, the enterprise, the government and the public embody a Pareto efficiency feature under a special condition, which is an inevitable reflection under a reasonable game condition. In the whole process of the game, the enterprise, the government and the public will naturally achieve the optimization of resource allocation. That is, when each party improves its own conditions, it meets its own requirements and at the same time tries to reduce the damage to the interests of others, or ensure that the interests of other parties will not suffer great losses, and at the same time achieve the optimal allocation of resources. Therefore, only when enterprises adopt environmental protection reconstruction, the government takes strict supervision measures, and the public actively participates in supervision, can the situation of the three parties be improved, so as to maximize the utility of social resources and reduce the occurrence of NIMBY conflicts.

## 5 Discussion and suggestions

On the basis of evolutionary game theory, this paper constructs the enterprise avoid polluting adjacent facilities reconstruction regulation avoid polluting adjacent facilities in the treatment of the enterprise, the local government and the public tripartite game theory gains matrix, avoid polluting adjacent facilities enterprise are analyzed in detail, the local government and the public to take the influence of different strategy combinations of regulatory governance. Through numerical experiment and simulation analysis of the game model, this paper draws the following conclusions:

1. Strategies of polluting NIMBY facilities enterprises, local governments and the surrounding public are different under different stability conditions. When the system is in main tripartite game don’t governance situation, can’t by increasing environmental modification fines and government regulation potential benefits, improve the polluting enterprises are not adjacent to avoid facility reconstruction potential losses, reduce public participation of environmental cost and the way of improving the long-term earnings, respectively, to promote enterprise avoid polluting adjacent facilities, the government and the public take active strategy, make the system to the x→1, y→1, z→1 the ideal state of evolution.
2. In the deterministic parameter selection, avoid polluting adjacent facilities main enterprises, local governments and the public any game strategy evolution speed will be affected by their own choice of the influence of the rate and the other two main strategy selection, but no matter how change, x, y, z values will change the final game group policy decision.
3. When the parameter value is determined, pollution-type NIMBY facility enterprises, local governments and the surrounding public can achieve the Pareto optimal solution, make full use of social resources, maximize their own interests and promote the harmonious development of society under the strategy (adopt environmental protection reconstruction, strict supervision and participation in supervision).

According to the above research conclusions, in order to promote environmental protection reconstruction of polluting NIMBY facilities enterprises, the following suggestions are put forward:

1. Whether polluting NIMBY facilities enterprises attach importance to the investment in environmental protection reconstruction depends to a large extent on the fines imposed by the government on polluting NIMBY facilities enterprises that do not attach importance to environmental protection reconstruction, the investment cost of environmental protection reconstruction of enterprises and the probability of strict supervision by the government on polluting NIMBY facilities enterprises. Considering the local government fines is avoid polluting adjacent facilities to purify sewage technology enterprise mainly binding, so you need to grasp of local government to the enterprise of rewards and punishments, through comprehensive legal and administrative means, avoid serious neglect environmental protection into the polluting of adjacent facilities enterprise production, closed rectify punishment, increase the intensity of punishment for illegal enterprises. At the same time, on the premise of implementing the "polluter pays principle", according to the local financial capacity, the polluting NIMBY facilities enterprises are encouraged to upgrade their equipment, such as tax exemption system, public funding subsidies, etc. Under the guidance of the incentive system, enterprises will pay more attention to the balance between environment and economy, which is conducive to the long-term development of society.
2. The choice of strict supervision strategy by local government mainly depends on the cost of supervision, the income of strict supervision, and the fine of negligent investment in environmental protection. In order to reduce the cost of strict supervision by local governments and increase the benefits of strict supervision, we should actively build a three-part linkage supervision mode with local governments as the leading body, polluting NIMBY facilities enterprises as the main body and the public as the participants. On the one hand, local governments should actively play a leading role, make full use of internal resources and information, mobilize experts and scholars, and offer professional opinions for environmental protection reconstruction of polluting NIMBY facilities enterprises; On the other hand, public media should actively publicize the necessity of public participation in supervision, improve public awareness of environmental protection, rationally utilize the hidden supervision function of the public, and promote environmental protection reconstruction of polluting NIMBY facilities enterprises.
3. The choice of strategic behavior of public participation in supervision depends on the cost of supervision reporting, the reward for civic supervision reporting and the long-term benefits. This indicates that the cost of public supervision and reporting can be reduced and the supervision and participation mechanism of the public can be improved through the establishment of a variety of smooth complaint channels (including network platform, complaint telephone, etc.). In addition, public individuals and environmental organizations participating in supervision can be encouraged and given appropriate rewards to increase their participation benefits and reduce participation costs, which can enhance the public’s motivation to participate in the supervision process of polluting NIMBY facilities enterprises.

## 6 Conclusion

### 6.1 Main findings

The reconstruction project of polluting NIMBY facility enterprises can play an important role in alleviating the NIMBY conflict among the surrounding public. However, NIMBY companies, which aim to make a profit, do not adopt the initiative to make green improvements. Therefore, it is essential to understand the factors that influence the active participation of the public, the government and NIMBY enterprises and how to improve the active participation of these three parties. The main research results are as follows:

1. In the game of environmental protection reconstruction of NIMBY enterprises, the three parties finally reach a balance state (the NIMby enterprise adopts environmental protection reconstruction, the government strictly supervises, and the public participates in supervision) to achieve the optimal Pareto efficiency and the optimal allocation of resources. Public participation in supervision plays an important role in promoting environmental reconstruction of NIMBY enterprises.
2. NIMBY enterprises, the government and the public are sensitive to changes in influencing factors. Increasing the amount of fines and promoting the potential benefits of government strict supervision can promote the government’s choice of strict supervision strategy; Reducing the cost of public participation and increasing the long-term benefits can promote the public participation in the supervision of the strategic choice; Increasing the potential loss of the NIMBY enterprises not to carry out environmental reconstruction can promote the strategic choice of the NIMBY enterprises to carry out environmental reconstruction.
3. The decision of public participation in supervision is not only affected by the cost of public supervision, but also by the government’s incentives and long-term benefits. To make the public actively participate in supervision, the government should not only provide appropriate incentives, but also let the public know the long-term benefits of supervision.

### 6.2 Theoretical implications

This study can provide new ideas for the government to alleviate NIMBY conflicts. This study has the following contributions to the existing literature:

1. This paper expands the research on the related fields of NIMBY conflict, and adds the public to the supervision system of NIMBY conflict, which provides a new Angle for getting rid of the dilemma of NIMBY.
2. This paper uses evolutionary game analysis to establish an evolutionary game model for the reconstruction of polluting NIMBY facilities, and analyzes the evolutionary mechanism of strict government supervision, environmental reconstruction by NIMBY enterprises, and public participation in supervision and governance. This model enriches the existing literature and shows that public participation can promote NIMBY enterprises to carry out environmental reconstruction and formulate reasonable and effective policies for the government to alleviate NIMBY conflicts.
3. Obtain the Pareto optimal solution by quantifying the interests of the three parties. The final results show that the three parties can optimize the allocation of resources and reduce the occurrence of NIMBY events when they adopt the strategy (environmental protection reconstruction by NIMBY enterprises, strict government supervision, and public participation supervision).

### 6.3 Practical implications

Through the analysis of the three-way game relationship between the government, the public and NIMBY enterprises, and the simulation results of each scenario, this paper has the following practical significance: Because NIMBY conflict is not conducive to social stability and hinders economic development, the government is facing huge challenges. By constructing a three-way linkage supervision mode with local government as the leader, NIMBY enterprises as the main body and public participation, the resistance movement of the surrounding public can be overcome. At the same time, under the premise of full utilization of resources and protection of the interests of all parties, the optimal strategy is proposed for the government, the public and NIMBY enterprises, providing an effective way to alleviate NIMBY conflicts.

### 6.4 Future work

There are some limitations in the research process of this paper: a numerical simulation is used, and certain assumptions are made when setting parameters, but the actual situation is far more complex than the simulation model. Besides, this paper simplifies the relationship between stakeholders, and the setting of influencing factors is not complete. In the next research center, influencing factors can be innovated. In addition, parameter values can be determined by analyzing actual cases to make the results more reliable.
